# Incidence of Clavicular Rhomboid Fossa in Northeastern Thais: An Anthropological Study

**DOI:** 10.1155/2016/9298043

**Published:** 2016-08-28

**Authors:** Ailadda Kaewma, Apichakan Sampannang, Panya Tuamsuk, Jaturat Kanpittaya, Sitthichai Iamsaard

**Affiliations:** ^1^Department of Anatomy, Faculty of Medicine, Khon Kaen University, Mitraparp Road, Khon Kaen, Thailand; ^2^Department of Radiology, Faculty of Medicine, Khon Kaen University, Mitraparp Road, Khon Kaen, Thailand; ^3^Center for Research and Development of Herbal Product, Faculty of Pharmaceutical Science, Khon Kaen University, Khon Kaen, Thailand

## Abstract

The rhomboid fossa of clavicle is used to determine the age and sex in anthropology and forensic sciences. The variant types of rhomboid fossa on inferior surface have been reported in many races except in Thais. This study therefore was aimed at classifying the types of the rhomboid fossa in Northeastern Thais. The identified 476 Northeastern Thais dried clavicles (270 males and 206 females) were observed and recorded for the types of rhomboid fossa. The results showed that Thai-rhomboid fossa could be classified into 4 types: Type 1: smooth; Type 2: flat; Type 3: elevated; and Type 4: depressed, respectively. The incidences of rhomboid fossa were as follows: Type 1: 0.21%; Type 2: 19.75%; Type 3: 76.26%; and Type 4: 3.78%, respectively. Additionally, it was found that the percentage of Type 4 (11.84%) was much greater than that of female (1.94%) compared to other types. This incidence of rhomboid fossa types especially Type 4 may be a basic knowledge to be used in sex identification. The high incidence of rhomboid fossa in both sexes of Northeastern Thai clavicles was Type 3 (elevated type).

## 1. Introduction

The skeletal bone remains of unidentified human can be found in many places around the world including Thailand. Skeletal remains are available for forensic identification such as sex, age, stature, and ancestry [1]. They were used for sex estimation such as pelvis [2], skull [3], upper and lower limb bones [4, 5], sternum [6], patella [7], foot bones [8], and clavicles [9–12]. For clavicle, various parameters including length, mid-shaft circumference [9–11], sternal end, acromial end [13, 14], and rhomboid fossa [15, 16] have been used to identify sex dimorphism in anthropology and forensic sciences.

In the literatures, the rhomboid fossa of clavicle, an area of the inferior surface of the sterna end, could be present as the impressions, tuberosities, depressions, or fossa [17–20]. Since the rhomboid fossa is attached by costoclavicular or rhomboid ligament, it is generally called “impression for costoclavicular ligament.” In Indians, Jit and Kaur [21] have reported that the rhomboid fossa between males and females was not different. In previous studies, however, the incidence of clavicular rhomboid fossa in American and Brazilian males was higher than that of females [15, 16]. In Thai population, the incidence and anthropological study of the clavicular rhomboid fossa have never been reported. Therefore, this study aimed to classify the types and provide the incidence of the rhomboid fossa's types investigated in Northeastern Thai dried clavicles.

## 2. Materials and Methods 

The 476 identified clavicles (left and right sides) from Northeastern Thai skeletons (270 males and 206 females) from KKU Osteological Collection Unit, Department of Anatomy, Faculty of Medicine, Khon Kaen University, were used in the study. The ages were ranged from 19 to 100 years. In sample selection, the incomplete clavicles such as fractured and plate-fixation clavicles were excluded. In observations, the inferior surface of the sterna end of individual clavicle was carefully investigated and the presence of types of the rhomboid fossa of clavicular bones was recorded. All results were calculated for the percentage of each type that was observed and further compared between males and females. In the comparison of all data, the significant differences between left and right clavicles were determined by Wilcoxon and Student's *t*-test using SPSS statistics. *P* < 0.05 was set as the level of statistical significance. As for the necessary human ethical clearance, this study was approved from the Office of The Khon Kaen University Ethics Committee for Human Research (the human ethic number HE581460).

**Figure 1:**
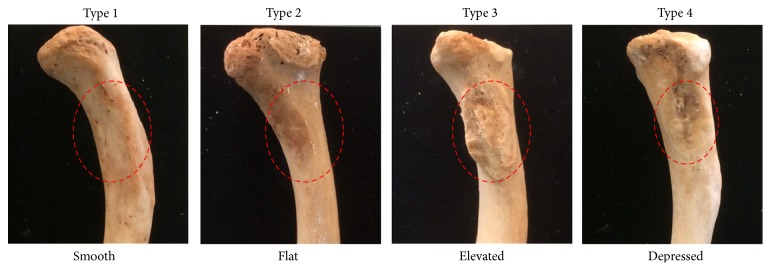
The representative photographs showing different types of rhomboid fossa observed in Northeastern Thai clavicles. They could be classified into 4 types: smooth (Type 1), flat (Type 2), elevated (Type 3), and depressed (Type 4), respectively.

In this study, the rhomboid fossa of 476 dried clavicles from Thai skeletons could be classified into 4 types based on Bhat and coworkers [17] as shown in Figure 1. Type 1 was smooth type showing no oval line or nodule or tubercle on inferior surface of the clavicular sterna end (Figure 1). Type 2 was flat type showing only oval line and no fossa with small nodules (Figure 1). Type 3 was elevated type showing many rough tubercles (Figure 1). Type 4 was depressed type showing oval fossa under inferior surface of the sterna end of clavicle (Figure 1).

## 3. Results

Table 1 shows incidence of 4 types of rhomboid fossa investigated in this study. The incidence showed that the percentages of clavicular rhomboid fossa observed in Northeastern Thais were as follows: smooth (Type 1): 0.21%; flat (Type 2): 19.75%; elevated (Type 3): 76.26%; and depressed (Type 4): 3.78%, respectively (Table 1). In males, it was found that the percentages of Types 1, 2, 3, and 4 were 0.74%, 35.55%, 151.85%, and 11.84%, respectively. Of females, the incidences of Types 1, 2, 3, and 4 were 0%, 44.65%, 153.40%, and 1.94%, respectively (Table 1). Individually, the incidences of Types 1, 2, 3, and 4 on left male clavicles were 0%, 23.70%, 68.15%, and 8.14%, respectively, whereas those on right male clavicles were 0.74%, 11.85%, 83.70%, and 3.70%, respectively (Table 1). In individual side of female clavicles, the incidences of Types 1, 2, 3, and 4 on left female clavicles were 0%, 24.27%, 75.73%, and 0%, respectively, whereas those on right male clavicles were 0%, 20.38%, 77.67%, and 1.94%, respectively (Table 1). In comparison, there was no significant difference between left and right sides of each rhomboid fossa type. In males, Type 3 of right side tended to be increasing whereas Type 2 at the same side tended to be decreasing (Table 1). In contrast to males, the percentages between right and left sides of individual type were not significantly different. Significantly, Type 3 of clavicular rhomboid fossa in males and females was found to be the highest as compared to the rest of the types (*P* < 0.005) (Table 1). Moreover, it was found that Type 4 of rhomboid fossa in males was significantly higher than that of females (*P* < 0.05).

## 4. Discussion

The present study has demonstrated the types and incidence of rhomboid fossa of dried clavicles in Thais for the first time (Figure 1). Previous studies have reported only two types (depressed and smooth) of clavicular rhomboid fossa in many populations such as Greeks [13], Americans [15], Brazilians [16], North Karnataka [17], North Indians [21], and Greeks [22]. In our observations (Figure 1), the types of rhomboid fossa were similar to that investigated in Indian clavicles which were classified into 4 types (smooth, flat, elevated, and depressed) [17]. It is possible that the lifestyles of these two populations are similar such as agricultural occupations in rural areas that might affect the rhomboid fossa development. In a radiological study, the high incidence (80%) of excavated type rhomboid fossa (corresponding to Type 4, depressed, in this study) on the dominant hand (right side) has been observed in Greece [22]. This was assumed to support the mechanical force of fossa formation. Similar to the Indians, this high incidence of Type 4 was found approximately in 67% [17]. In contrast to Greece and Indians, Type 4 fossa in Northeastern Thais was approximately 3.78% (Table 1). It seemed that Type 4 of rhomboid fossa in Greece and Indians is dominant on the right clavicle of males [17, 22]. In contrast to the Greece and Indians, Type 3 (elevated type) of rhomboid fossa was found to be of high incidence in both sexes but not different between right and left sides (Table 1). Therefore the clavicular fossa formation of Type 3 of Northeastern Thais could still not be explained as mechanism theory like other races. Interestingly, Type 2 and Type 4 observed in this study can be used to identify males or females. Type 2 of males (35.55%) was lesser than that of females (44.65%) by around 1.25-fold. In contrast, Type 4 of males (11.84%) was greater than that of females (1.94%) by around 6.1-fold. However, the percentages of these two types were still limited in validation. Compared to a previous study [17], we found that Type 3 (elevated) in Thai race (76.26%) was higher than that of Indian (7.32%), whereas Type 1 (smooth) in Indians (11.38%) was greater than Northeastern Thais (0.21%). It is possible that the different lifestyles of these populations might cause the varying formations of individual rhomboid fossa. Since these clavicles observed in recent study were mostly old and had very wide age distribution, the analysis of age variation was limited. In conclusion, the rhomboid fossa of Northeastern Thais can be classified as 4 types (smooth, flat, elevated, and depressed). The high incidence of rhomboid fossa in both sexes is Type 3 (elevated type).

## Figures and Tables

**Table 1:** Incidence of rhomboid fossa of clavicle between male and female.

Genders	Numbers	Ages (years) (mean ± SD)	Types of rhomboid fossa of clavicle							
			Type 1: smooth		Type 2: flat		Type 3: elevated		Type 4: depressed	
			Left	Right	Left	Right	Left	Right	Left	Right
Males	*N* = 270 (Lt = 135, Rt = 135)	15–86 (~61 ± 16.10)	0 (0%)	1 (0.74%)	32 (23.70%)	16 (11.85%)	92 (68.15%)	113 (83.70%)	11 (8.14%)	5 (3.70%)
			Total = 0.74%		Total = 35.55%		Total = 151.85%^*∗∗*^		Total = 11.84%^*∗*^	

Female	*N* = 206 (Lt = 103, Rt = 103)	26–94 (~60 ± 13.38)	0 (0%)	0 (0%)	25 (24.27%)	21 (20.38%)	78 (75.73%)	80 (77.67%)	0 (0%)	2 (1.94%)
			Total = 0%		Total = 44.65%		Total = 153.40%^*∗∗*^		Total = 1.94%	

*Total*	*N* = 476	60.5 ± 14.74 (average)	1 (0.21%)		94 (19.75%)		363 (76.26%)^*∗∗*^		18 (3.78%)	

^*∗∗*^ *P* < 0.005 (Type 3 versus Type 1, 2, or 4).

^*∗*^ *P* < 0.05 (males versus female).
